# The gut–lung axis in early life: microbiome-associated mechanisms and clinical relevance

**DOI:** 10.1183/16000617.0147-2026

**Published:** 2026-09-23

**Authors:** Lara Klischke, Alexandros Rahn, Ingmar Fortmann, Marie-Theres Dammann, Isabell Ricklefs, Fumi Sugihara, Kirsten Glaser, Claus Klingenberg, Christoph Härtel, Sabine Pirr

**Affiliations:** 1 Department of Pediatric Pneumology, Allergology and Neonatology, Hannover Medical School, Hannover, Germany; 2 Department of Pediatrics, University of Lübeck Medical School, Lübeck, Germany; 3 Department of Pediatrics, University Hospital Würzburg, Würzburg, Germany; 4 Division of Neonatology, Department of Women's and Children's Health, University of Leipzig Medical Center, Leipzig, Germany; 5 Research Group for Child and Adolescent Health, Faculty of Health Sciences, UiT – The Arctic University Norway, Tromsø, Norway; 6 Department of Pediatrics and Adolescence Medicine, University Hospital of North Norway, Tromsø, Norway; 7 Cluster of Excellence RESIST 2155 – Resolving Infection Susceptibility, Hannover Medical School, Hannover, Germany

## Abstract

The early-life microbiome is increasingly recognised as a contributor to immune maturation and respiratory health. Early-life microbial colonisation is highly dynamic and shaped by multiple factors. Recently, the gut–lung axis has emerged as a framework describing bidirectional communication between the intestinal microbiota and the respiratory system *via* microbial metabolites, immune mediators and neuroimmune pathways. Although current evidence is largely derived from studies of bacterial communities, emerging data suggest that other microbial components, including fungi and viruses, may also contribute to gut–lung interactions. This review synthesises current mechanistic and clinical evidence on the gut–lung axis with a specific focus on neonatology. We discuss how early microbiome composition may influence immune regulation, lung development and inflammatory response. Associations between intestinal microbiota alterations and major neonatal morbidities, including bronchopulmonary dysplasia, are discussed, as well as emerging evidence linking early-life microbial imbalance to long-term respiratory outcomes such as recurrent infections and asthma. We further examine emerging translational opportunities, including microbiome- and metabolome-based biomarkers for early risk stratification, and microbiota-targeted interventions. Attention is given to the strengths and limitations of the current evidence base, including the predominance of observational human studies and preclinical animal models. Overall, available evidence suggests that the developing microbiome represents a potentially important mediator of gut–lung communication in early life. However, substantial gaps remain regarding causality, underlying mechanisms and clinical applicability. Further longitudinal and interventional studies are needed to define the role of microbiome-targeted strategies in improving neonatal and long-term respiratory outcomes.

## Shareable abstract

**Early-life microbiome shapes immune maturation and may influence respiratory health. Alterations have been associated with respiratory disease, as BPD and RTIs. The gut–lung axis offers potential new targets for prevention and personalised neonatal care.** https://bit.ly/3S36wtT

## Introduction

The human body is a complex and dynamic habitat, hosting diverse microbial ecosystems across surfaces such as the skin, oral cavity, respiratory, genitourinary and gastrointestinal tract [1]. These microbial populations, collectively referred to as the human microbiota, are shaped by the unique characteristics of their host environment and constitute a distinct metagenome and associated functional repertoire, the microbiome [1, 2]. The composition and functional capacity of these microbial communities are increasingly recognised as critical determinants of health throughout the human lifespan, from infancy to adulthood [3, 4]. The initial colonisation of the microbiome begins at birth, representing the first encounter with maternal and environmental microbes, and is subsequently shaped by numerous endogenous and exogenous factors, including mode of delivery, nutrition, infection and exposure to antibiotics [5–10]. Disruptions in the early development of these microbial ecosystems can have lasting consequences for systemic immunity and organ function, including increased susceptibility to respiratory infections and impaired lung maturation [8, 11].

Over the past decade, the concept of the gut–lung axis has emerged, describing bidirectional interactions between the intestinal microbiome and pulmonary physiology [11–13]. Current evidence on the early-life gut–lung axis is mainly derived from studies of bacterial communities, largely based on 16S rRNA sequencing approaches [14, 15]. While the terms microbiota and microbiome encompass a broader range of microorganisms including fungi, viruses, archaea and their functional interactions with the host, these components remain comparatively understudied in neonatal populations [16, 17]. Therefore, this review primarily focuses on bacterial microbiota while highlighting emerging evidence regarding other microbial kingdoms where available. Experimental and clinical studies suggest that intestinal microbes may influence pulmonary physiology through several proposed mechanisms, including microbial metabolites, immune modulation and, in some settings, microbial migration [18–20]. In preterm infants, the presence of a gastric tube has been associated with bidirectional microbial exchange between the lung and the gastrointestinal tract [21, 22]. This may result from increased aspiration of oropharyngeal microbes, reflux with secondary aspiration and direct transfer of bacteria into the stomach during tube insertion or by partial opening of the gastro-oesophageal sphincter, facilitating microbial movement between compartments [23].

Alterations in the establishment of the intestinal microbiome after birth have been associated with an increased frequency and severity of respiratory tract infections, as well as with chronic diseases such as bronchopulmonary dysplasia (BPD) [8, 24–26] and asthma [20, 27]. For BPD in particular, this association may at least partly reflect shared upstream drivers such as illness severity and treatment intensity rather than a direct causal pathway [28]. Conversely, beneficial microbiota constellations have been identified and may serve as future targets for therapeutic interventions, including probiotics, prebiotics and targeted nutritional strategies [29, 30].

Given the growing body of observational evidence linking early-life microbiota composition to pulmonary outcomes, the gut–lung axis has emerged as a candidate framework for understanding both acute neonatal disease and long-term respiratory health, although causal evidence in humans remains limited [14]. Therefore, this review synthesises current clinical and experimental evidence on the significance of gut–lung crosstalk, with a particular emphasis on neonates and children. We first outline the development and composition of the gut microbiota, then examine the mechanistic pathways through which intestinal microbes shape lung immunity and finally discuss disease associations, clinical implications and emerging translational opportunities.

## Methods

This article was conducted as a narrative review. A structured literature search was conducted using PubMed to identify relevant publications on the gut–lung axis in neonates and infants. Search terms included combinations of “gut–lung axis”, “microbiome”, “preterm infants”, “bronchopulmonary dysplasia”, “necrotizing enterocolitis”, “late-onset sepsis”, and “respiratory infections”. Both clinical and experimental studies were considered, with emphasis on paediatric cohort studies, mechanistic insights from animal models and relevant systematic reviews. Additional articles were identified through manual screening of reference lists. Studies were selected based on relevance to early-life microbiota development, immune mechanisms and respiratory outcomes. As a narrative review, no formal systematic review methodology, predefined protocol or quantitative assessment of study quality was applied. Throughout this review, we explicitly distinguish associations from causal evidence and indicate where findings derive from animal models rather than human studies, with a dedicated appraisal of methodological limitations.

## Development and composition of intestinal microbiome

The human gut microbiome is a complex and dynamic ecosystem shaped by multiple factors during the first years of life [2, 11, 31]. It comprises a diverse array of microorganisms, including bacteria, viruses and fungi, of which bacteria make up the largest proportion [31]. While the composition varies significantly between individuals, the four most dominant phyla of bacteria that constitute the human microbiota are *Bacteriodia*, *Firmicutes*, *Actinobacteria* and *Proteobacteria* [32]. Among the various factors influencing the development of the gut microbiota, the mode of delivery has been identified as particularly significant in terms of initial colonisation patterns. Neonates delivered *via* caesarean section typically exhibit delayed colonisation of the intestine by *Lactobacillus*, *Bifidobacteria* and *Bacteroides* compared to those delivered vaginally [15, 33]. Age and breast milk feeding represent established determinants of intestinal bacterial colonisation. Breast milk-derived S100A8/A9 and fecal S100A8/A9 (also referred to as calprotectin) causally shapes gut microbiota development in murine models, while corresponding human data so far remain associative [15, 34]. Breastfeeding promotes high levels of human milk oligosaccharide (HMO)-adapted *Bifidobacterium* taxa*,* particularly *B. infantis*, *B. longum* and *B. breve* [15, 35]. Formula feeding can also support *Bifidobacterium* colonisation, but typically with a more diverse species profile that additionally includes non-HMO-specialised taxa otherwise seen in adults, unless formulas are supplemented with HMOs, which shifts the formula-fed microbiome closer to that of breastfed infants [36]. During the transition to complementary feeding, HMO-adapted *Bifidobacterium* taxa are progressively complemented or replaced by other taxa, including a distinct *B. longum* clade specifically adapted to weaning-associated substrates, marking an important milestone in the maturation and diversification of the intestinal microbial community [15, 35]. The gut microbiota maturation is generally conceptualised in three main phases: the developmental phase (first year of life), the transitional phase (ages 1–3 years) and the stable phase (age 3 years onwards) [15, 37, 38]. Key influencing factors of the infant's microbiome are family and household contacts as well as strain-to-strain transmission in nursing care facilities [37, 38]. However, the microbiota can still be influenced after age of 3 years by external factors such as diet, infections and medications [37]. Preterm infants are more susceptible to gut microbiota instability, often compounded by additional risk factors such as antibiotic exposure, permanent exposure to hospital-associated microbes and delayed enteral feeding, which predispose them to intestinal dysbiosis [37, 39]. Intestinal dysbiosis in this population precedes and is associated with increased susceptibility to necrotising enterocolitis (NEC) [24, 25, 40, 41] and late-onset neonatal sepsis (LONS) [42], and has also been observed in human cohorts in association with BPD [24]. However, a causal contribution to BPD has so far only been demonstrated in animal models of microbiota manipulation, not in neonates [25]. Beyond the neonatal period, intestinal dysbiosis has also been linked to long-term conditions such as inflammatory bowel disease and allergic disorders [27, 43]. Understanding the factors that shape early-life microbial colonisation is therefore essential for identifying high-risk infants and developing strategies to modulate the gut microbiota for improved short- and long-term health outcomes.

### Beyond bacteria: emerging roles of the mycobiome and virome

Although most evidence on the early-life gut–lung axis derives from studies of bacterial communities, the microbiome also encompasses fungi, viruses and other microorganisms that may contribute to immune development and respiratory health. Fungal colonisation begins early in life and evolves with gestational age, forming dynamic interactions with bacterial communities and the host immune system [44–46]. Beyond their relatively low abundance, fungi appear to play important ecological roles in maintaining microbial network stability and regulating host immune responses [16, 47]. Emerging experimental evidence suggests that fungal communities may directly influence gut–lung communication. In neonatal mouse models, fungal microbiota have been shown to modulate hyperoxia-induced lung injury, while alterations in intestinal microbial communities can affect pulmonary inflammation and immune responses [26, 48, 49]. In contrast, the contribution of the neonatal virome remains poorly understood. However, bacteriophages and eukaryotic viruses are increasingly recognised as important regulators of microbial community structure and host immunity, highlighting the need for future multi-omics approaches that integrate bacterial, fungal, viral and metabolic datasets to better characterise microbiome-mediated gut–lung interactions in early life [17].

## Mechanisms of the gut–lung interaction

**FIGURE 1:**
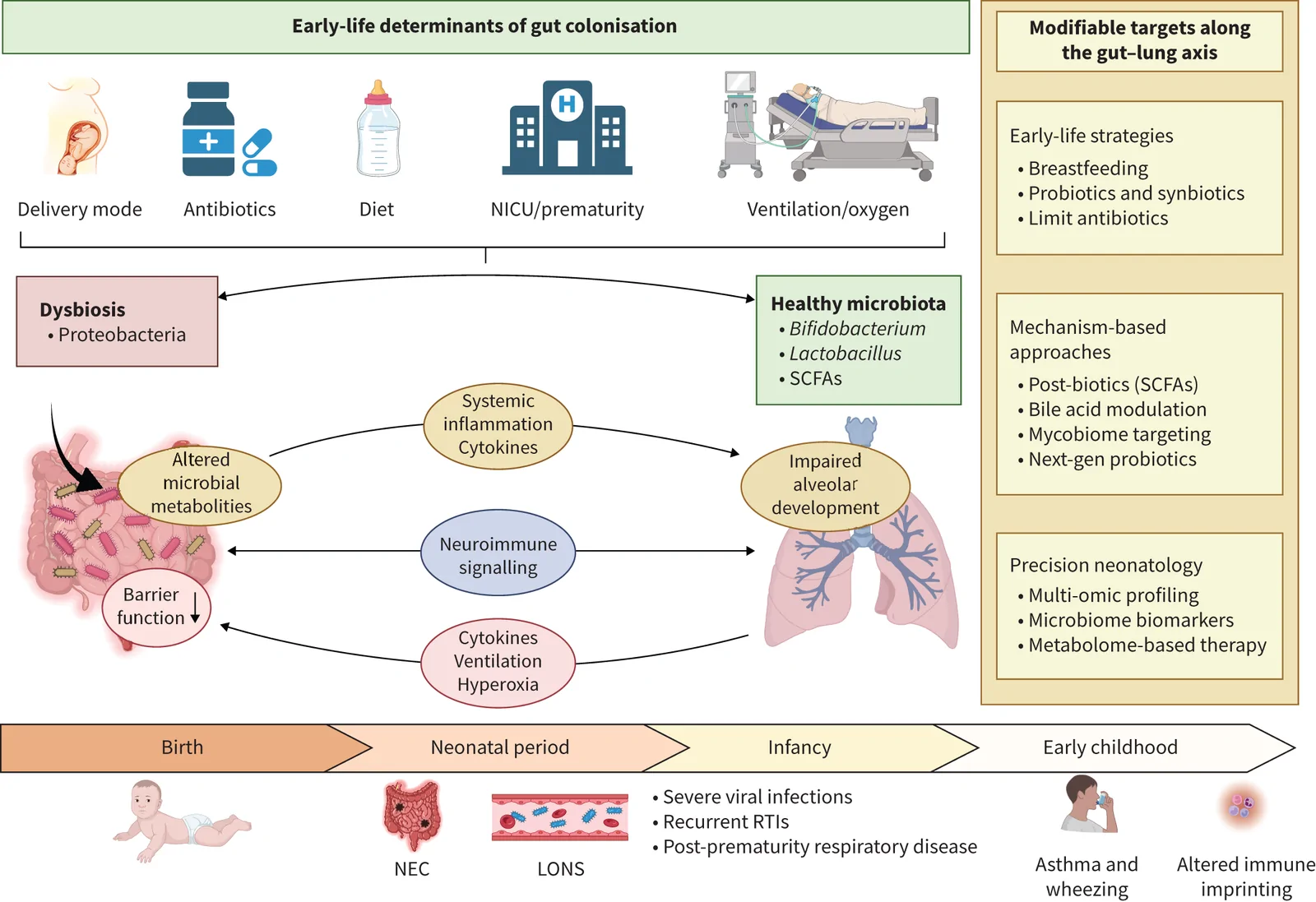
Early-life determinants of gut colonisation and gut–lung crosstalk. Schematic representation of how perinatal exposures, including delivery mode, antibiotic use, breast milk (human milk oligosaccharides), prematurity/neonatal intensive care unit (NICU) stay and ventilation/oxygen therapy, shape early gut microbial assembly. These factors promote either a healthy microbiota (*e.g.* *Bifidobacterium*, *Lactobacillus*, short-chain fatty acids (SCFAs)) or dysbiosis (*e.g.* Proteobacteria predominance). Dysbiosis is associated with altered microbial metabolites, impaired intestinal barrier integrity and systemic inflammation. Through immune and neuroimmune signalling pathways, including cytokine responses influenced by ventilation and hyperoxia, gut-derived signals modulate alveolar development and pulmonary immunity. These gut–lung interactions contribute to respiratory and systemic outcomes across early life, including bronchopulmonary dysplasia (BPD), necrotising enterocolitis (NEC), late-onset neonatal sepsis (LONS), recurrent respiratory tract infections (RTIs), wheezing and asthma. The figure also highlights modifiable targets along the gut–lung axis, including breastfeeding, probiotic/synbiotic strategies, antibiotic stewardship, postbiotics (*e.g.* SCFAs), bile acid modulation, mycobiome targeting, next-generation (next-gen) probiotics and precision neonatology approaches (multi-omic profiling and microbiome-informed therapies).

Communication between the intestine and distant organs, including the lung, is mediated predominantly through two interrelated pathways, namely soluble molecular mediators and neuroimmune cell–cell interactions [50]. The first pathway involves microbiota-derived metabolites and immune mediators, such as short-chain fatty acids (SCFAs), amino acid derivatives including tryptophan metabolites, bacterial cell wall components and cytokines, which collectively enable systemic signalling beyond the gut [11, 51]. Among these, SCFAs, primarily acetate, propionate and butyrate, are generated through anaerobic fermentation of dietary fibres and represent key effectors of host–microbe interactions [52]. SCFAs exert their biological effects through activation of G protein-coupled receptors and inhibition of histone deacetylases, thereby modulating transcriptional programs relevant to immune regulation and cellular metabolism [53]. Experimental models highlight their essential role in systemic immune homeostasis, as germ-free mice lacking SCFA receptors exhibit impaired immune and microglial development [54]. Gut microbiota-derived SCFAs promote the expansion and functional stability of regulatory T-cells (Tregs), reinforcing systemic anti-inflammatory immune responses [55, 56]. Furthermore, within the intestine, butyrate serves as a primary energy source for colonocytes and reinforces epithelial barrier integrity, collectively fostering an anti-inflammatory milieu [55, 57–59]. Importantly, SCFAs can enter the systemic circulation and reach peripheral organs, including the lung, where they enhance antimicrobial defences and support effective pathogen clearance, underscoring their role in gut–lung immune crosstalk [54, 60]. In addition, alterations in microbial amino acid metabolism represent a potential mechanistic link within the gut–lung axis. Hippurate is a host–microbiota co-metabolite formed when intestinal bacteria convert phenylalanine to benzoic acid, which is subsequently conjugated in the liver. Reduced hippurate levels are associated with intestinal dysbiosis and decreased microbial diversity in large human cohorts [61, 62]. Changes in phenylalanine-derived microbial metabolites may contribute to disease severity by modulating immune responses beyond the gut compartment, thereby linking intestinal metabolic activity to pulmonary outcomes (figure 1).

The second major communication pathway relies on direct neuroimmune interactions, integrating immune cells with the peripheral and enteric nervous systems. Concurrently, neural circuits involving the vagus nerve, dorsal root ganglia and the enteric nervous system dynamically modulate immune activity. Neural mediators can either amplify or suppress Treg function, thereby fine-tuning immune responses across mucosal sites [63]. Neuromedin U (NMU), released by cholinergic neurons, exemplifies this interaction by stimulating group 2 innate lymphoid cells (ILC2s) and inducing type 2 cytokine production, linking neural signalling to mucosal immunity and inflammation [63].

Emerging evidence indicates that the lung is not only a target organ but also an active regulator of intestinal homeostasis. Pulmonary inflammation can trigger systemic inflammatory responses that secondarily affect the gastrointestinal tract [64, 65]. Pro-inflammatory mediators released in the lung, including cytokines such as interleukin (IL)-6 and tumour necrosis factor (TNF)-α, may enter the systemic circulation and impair intestinal epithelial integrity by disrupting tight junctions, thereby increasing intestinal permeability (figure 1) [66–68]. In addition to inflammatory signalling, respiratory disease and its treatment can influence intestinal physiology through haemodynamic and metabolic pathways [66, 69–71]. Mechanical ventilation, fluctuations in oxygenation and exposure to hyperoxia may alter splanchnic perfusion and promote oxidative stress, both of which have been associated with impaired gut motility and alterations in the developing intestinal microbiota [71]. Furthermore, neuroimmune pathways, including vagal signalling, have been proposed as additional mediators of lung–gut communication [69, 70].

The depth of evidence supporting these mechanisms differs markedly once their broader research context is considered. SCFA-mediated immune effects are supported by an extensive and largely consistent literature spanning rodent, cell culture and some human studies [52, 53, 56]. The NMU–ILC2 pathway has likewise been independently confirmed by several research groups in mice [63, 72] and recently extended to human airway samples in adult asthma, where NMU receptor expression on sputum ILC2s increased after allergen challenge [73]. However, functional evidence remains predominantly from murine models and this pathway has not been studied in neonates. Hippurate is a well-established, repeatedly replicated marker of gut microbial diversity and metabolic health in large human cohorts [62] but this evidence concerns metabolic and cardiovascular outcomes in adults, not the lung. A link to pulmonary outcomes has so far only been suggested by a single study in preterm infants with BPD [74]. For all mechanisms discussed above, direct evidence specific to the neonatal gut–lung axis remains limited and the relevance of pathways established in nonpulmonary, adult or nonhuman contexts to early-life lung disease should be considered hypothesis-generating rather than established.

In summary, a tightly coordinated network of microbial metabolites, immune signalling pathways and neuroimmune interactions that collectively regulate systemic and pulmonary immune homeostasis underpins gut–lung crosstalk (figure 1) [50, 51, 53, 69, 71]. These foundational mechanisms provide a biological framework explaining how disturbances in gut microbial composition or function can propagate inflammatory signals to the lung, particularly during vulnerable developmental windows such as in early life and in preterm infants. A detailed understanding of these core interactions is essential for interpreting disease associations and serves as the mechanistic basis for translational strategies aimed at modulating gut-derived signals to prevent or attenuate lung inflammation and injury.

## Clinical evidence of gut–lung interaction

The concept of gut–organ crosstalk has long been recognised and advances over recent decades have increasingly delineated its clinical relevance, particularly with respect to interactions between the gut microbiota and the respiratory system [75–78]. Accumulating clinical and epidemiological evidence indicates that reciprocal communication between the gut and the developing lung, referred to as the gut–lung axis, plays an important role in shaping respiratory and systemic outcomes in early life. This interaction appears especially relevant in preterm infants, in whom immaturity of the intestinal barrier, immune system and lung coincides with critical windows of microbial and immune development and is further influenced by antibiotic exposure and intensive care interventions [18, 27, 43, 79]. Alterations in early gut microbial composition and metabolic function have been associated with major complications of prematurity, including BPD, NEC and LONS, which share overlapping inflammatory, vascular and metabolic pathways while they are strongly linked to adverse respiratory trajectories. Moreover, a systematic review of longitudinal cohort studies suggests that early-life gut dysbiosis is associated with respiratory tract infections (RTIs) and wheezing disorders extending into childhood, beyond the neonatal period [14]. We focus on BPD, LONS, NEC and RTI/long-term outcomes because these represent the conditions for which gut–lung axis involvement has been most extensively studied in preterm and term infants. The depth and quality of this evidence nonetheless varies considerably between outcomes, as detailed in the respective subsections below. General methodological limitations affecting this evidence base as a whole are summarised in the “Strengths and limitations of the current evidence” section. As this is a narrative rather than a systematic review, we do not report an exhaustive count of studies or included infants for each outcome; readers seeking quantitative synthesis are referred to the systematic reviews and meta-analyses cited throughout [14, 40, 80, 81].

### Bronchopulmonary dysplasia (BPD)

BPD is a heterogeneous and multifactorial disorder arising from recurrent injury to the immature lung and dysregulated repair processes driven by exposures such as infection, mechanical ventilation, and hyperoxia [82, 83]. Increasing evidence indicates that pulmonary development and inflammatory responses in preterm infants are not determined solely by local pulmonary insults but are also shaped by systemic influences, including signalling along the gut–lung axis [76–78]. The pulmonary microenvironment is particularly susceptible to microbiota-mediated effects, both from intrapulmonary and extrapulmonary sources [84, 85].

Distinct microbial communities colonise both the upper and lower respiratory tract and dynamically interact with host immune pathways [86, 87]. Several studies suggest an association between disturbances in the respiratory microbiome composition and BPD development [87–89]. However, the evidence remains limited and inconclusive, and it is unclear whether microbial imbalance drives inflammation or results from impaired postnatal immune maturation. Given that the gut microbiome represents the body's largest microbial reservoir, it may play a predominant role in shaping pulmonary immunity, potentially contributing to the persistent uncertainty surrounding the respiratory microbiome's role in infectious and noncommunicable lung diseases.

Preterm infants who develop BPD exhibit early and sustained gut microbiota dysbiosis, characterised by reduced microbial diversity, overrepresentation of opportunistic *Proteobacteria* (*e.g.* *Brevundimonas*) and depletion of beneficial commensals, changes that precede the onset of severe disease [24, 28, 90]. Beyond taxonomic alterations, disruptions in microbial metabolic pathways, including bile acid and SCFA signalling, have been identified as early biomarkers associated with BPD severity and systemic immune dysregulation [90]. In parallel, emerging evidence implicates that the neonatal gut mycobiome may also play a role in lung injury, with preclinical studies demonstrating that dysbiotic fungal colonisation exacerbates hyperoxia-induced lung pathology in a microbiota-transferable manner [20, 26].

Collectively, these mechanistic insights have spurred interest in microbiota-targeted therapeutic strategies. Experimental models demonstrate that probiotic or microbiota-directed interventions can attenuate lung inflammation, improve alveolarisation and reduce pro-inflammatory cytokine signalling through gut–lung crosstalk [29, 91–93]. However, clinical trials of probiotic supplementation in preterm infants have yielded inconsistent effects on BPD incidence, despite well-established benefits for NEC [94]. Beyond direct microbial supplementation, modulation of microbial metabolites and bile acid-microbiota signalling pathways represents a promising avenue for early risk stratification and therapeutic intervention in severe BPD [90]. Future large-scale longitudinal studies integrating microbiome, metabolome and immune profiling will be essential to translate gut–lung axis-based strategies into safe and effective therapies for extremely preterm infants.

### Late-onset neonatal sepsis (LONS)

Preterm infants are highly susceptible to LONS, a major contributor to morbidity and mortality in neonatal intensive care units (NICUs) [95–97]. Increasing evidence indicates that gut microbiota dysbiosis precedes and predicts LONS, with reduced diversity, overgrowth of *Proteobacteria* or *Staphylococcus*, and loss of beneficial anaerobes such as *Bifidobacterium* and *Lactobacillus* preceding bloodstream infection [42, 98–100]. Gut-derived pathogens and microbial products trigger systemic inflammation, characterised by elevated cytokines including IL-6, TNF-α and IL-1β, which not only propagate sepsis but also disrupt distal organ development including the lung [101, 102]. Cytokines and microbial products reaching the pulmonary vasculature stimulate alveolar macrophages and epithelial cells, leading to aberrant vascular endothelial growth factor- and transforming growth factor-β-mediated alveolar and vascular remodelling, impaired surfactant production, and recruitment of neutrophils and monocytes that exacerbate pulmonary tissue injury [24]. Experimental and mechanistic studies in sepsis models indicate that gut dysbiosis or antibiotic-driven microbiome disruption can amplify these responses by reducing regulatory microbial metabolites (*e.g.* SCFA) that normally suppress Toll-like receptor 4 (TLR4)/nuclear factor-κB (NF-κB) activation and maintain epithelial resilience, thereby linking intestinal microbial ecology to pulmonary immune homeostasis; this evidence, however, derives largely from adult and animal sepsis models rather than neonatal cohorts [103]. A longitudinal cohort study of preterm and term infants additionally found that gut and respiratory microbiota develop in a temporally coordinated manner across the first year of life, suggesting shared developmental regulation across mucosal sites [104]. Together, these observations support a model in which early gut microbial perturbations in preterm infants may contribute to systemic infection risk and, secondarily, to pulmonary complications *via* the gut–lung axis, although this link remains largely correlative in human neonates and has not been directly tested experimentally in this population [105].

### Necrotising enterocolitis (NEC)

NEC is a severe complication of prematurity and a leading cause of morbidity and mortality in very low birthweight infants [106–108]. Mechanistically, NEC pathogenesis is strongly associated with intestinal dysbiosis, wherein early shifts in microbial composition, characterised by overrepresentation of pathogenic *Proteobacteria* and reduced commensal *Firmicutes* and *Bifidobacteria*, precede disease onset and disrupt epithelial homeostasis [40, 109, 110]. Intestinal dysbiosis promote epithelial barrier dysfunction through direct cytotoxic effects, impaired tight junction integrity and modulation of mucosal immune signalling, resulting in exaggerated activation of pattern recognition receptors (*e.g.* TLR4) and downstream NF-κB-mediated pro-inflammatory cascades [111–113]. The consequent local inflammatory milieu, enriched in cytokines such as TNF-α, IL-6 and IL-1β, facilitates translocation of microbial products (lipopolysaccharide, peptidoglycan) into the systemic circulation, triggering systemic inflammation [111–115]. This systemic inflammatory response can extend to distant organs, including the lung, where circulating microbial products and cytokines perturb alveolar and vascular development, linking NEC-associated dysbiosis to biological pathways implicated in BPD [110, 116]. Experimental and clinical data further demonstrate that microbiota-directed interventions, most notably probiotics containing *Bifidobacterium* and *Lactobacillus* species, can restore microbial balance, enhance epithelial barrier function, and attenuate excessive TLR4–NF-κB signalling, thereby reducing the incidence and mortality of NEC [117–119].

### Respiratory immunity and respiratory tract infections (RTIs)

Growing evidence supports a role for the gut microbiota in shaping respiratory immunity *via* the gut–lung axis. In early life, when both the gut microbiome and the immune system are highly plastic, intestinal dysbiosis has been associated with increased susceptibility to RTIs, including findings that respiratory syncytial virus (RSV) disease severity in hospitalised infants is associated with differences in gut microbiota alpha and beta diversity at the time of infection [120]. In mice, gut-derived metabolites appear largely protective against respiratory infection. Acetate enhances antiviral defence against RSV by promoting type 1 interferon signalling and interferon-stimulated gene expression in lung epithelial cells *via* G-protein coupled receptor 43 activation [121] and *Bacteroides*-derived propionate contributes to protection against early-life bronchiolitis [122]. Conversely, reduced levels of intestinal SCFAs, including pentanoate and hexanoate, impair pulmonary type 2 innate lymphoid cell function and exacerbate inflammation induced by first breaths and respiratory infections [123]. Several murine studies demonstrate that the gut microbiota enhances resistance to bacterial and viral respiratory infections by modulating alveolar macrophage function, increasing pulmonary granulocyte–macrophage colony-stimulating factor production, and augmenting interferon-β-mediated antiviral immunity [124–126].

In human infants, evidence, largely derived from observational studies, has identified associations between specific gut microbial profiles and respiratory diseases. Recent shotgun metagenomic analyses provide support for early-life gut–lung interactions; a prospective cohort study demonstrated that higher gut microbiota alpha diversity during the first week of life and a microbial community dominated by *B. longum* were associated with reduced hospital admissions for severe viral lower RTIs during the first 2 years of life [127]. Interventional studies suggest that modulation of the infant gut microbiota is feasible; for example, probiotic supplementation in formula-fed infants resulted in enrichment of *B. breve*, reduction of *Klebsiella* and increased levels of microbial metabolites implicated in immune signalling and gut–lung communication, although this did not translate into a significant reduction in RTI incidence during the first year of life [30]. Notably, gut overrepresentation of *Klebsiella* has been linked to a pro-inflammatory immune phenotype and adverse systemic outcomes in neonates [128], underscoring the potential importance of early microbial balance. Early gut microbial composition may thus influence respiratory immune development and disease vulnerability, even though causal pathways and target cell populations, particularly within the airway epithelium, remain incompletely defined. Identifying early-life microbial and environmental factors that promote a health-supporting gut–lung axis may therefore offer novel strategies to reduce the burden of severe respiratory infections across the life course.

### Long-term respiratory consequences

Beyond neonatal morbidity, increasing evidence indicates that early-life perturbations of the gut–lung axis exert long-lasting consequences for respiratory health, extending into childhood and adolescence [4, 51, 77]. Epidemiological studies consistently identify preterm birth as an independent risk factor for wheezing disorders and asthma, with meta-analyses demonstrating a significantly higher asthma prevalence among individuals born preterm compared with term-born controls [80]. This association is likely multifactorial: preterm birth interrupts lung development during the canalicular and saccular stages, before alveolarisation is complete [129], and this structural immaturity, rather than microbiome-driven immune dysregulation alone, may account for much of the excess respiratory morbidity, particularly since preterm infants overall show lower rates of atopy and allergic sensitisation than term-born infants, suggesting that nonallergic mechanisms may underlie much of their excess asthma risk [130]. Seminal work by Arrieta *et al*. [131] demonstrated that reduced abundance of specific gut bacterial taxa and altered SCFA profiles in infancy preceded the development of childhood asthma, and that transfer of dysbiotic infant microbiota into germ-free mice increased susceptibility to allergic airway inflammation. These findings provide causal evidence that early gut microbiota composition shapes long-term pulmonary immune trajectories. Building on these findings, Budden *et al*. [132] proposed that impaired microbial-driven immune education along the gut–lung axis results in persistent dysregulation of T-helper (Th) 2 type and Th17 type immune responses, predisposing to chronic airway inflammation and asthma. In preterm populations, factors such as repeated antibiotic exposure, delayed microbial maturation and early inflammatory insults may further exacerbate maladaptive immune imprinting, amplifying lifelong respiratory risk [133–135].

Collectively, these data highlight the gut–lung axis as a compelling target for translational interventions, suggesting that early modulation of the gut microbiota may not only reduce short-term pulmonary morbidity but also represent a preventive strategy against acute and chronic respiratory diseases. Integrating longitudinal microbiome, metabolome and immune profiling in preterm and term birth cohorts could enable risk stratification, early identification of susceptible infants and personalised interventions, bridging mechanistic understanding to clinically actionable strategies.

## Strengths and limitations of the current evidence

Research on the early-life gut–lung axis provides increasing biological plausibility for bidirectional communication between the intestinal microbiome and the developing respiratory system. Mechanistic studies have identified several pathways, including microbial metabolites, immune cell trafficking and neuroimmune signalling, through which intestinal microorganisms may influence pulmonary immune responses and lung development [50, 54, 55]. However, the current evidence base has important limitations that warrant cautious interpretation. Although numerous studies have demonstrated associations between intestinal microbial composition and respiratory diseases such as BPD, respiratory infections and asthma, direct evidence supporting a causal role of the gut–lung axis in humans remains limited [14, 136, 137]. Many clinical studies are observational, involve relatively small and heterogeneous cohorts, and differ substantially regarding gestational age, antibiotic exposure, feeding practices, sampling time-points, outcome definitions, sequencing methodologies and bioinformatic pipelines [11, 46, 132, 138]. These methodological differences contribute to inconsistent findings and limit comparisons across studies. Moreover, alterations in the intestinal microbiome may reflect the severity of underlying illness or intensive care interventions rather than representing an independent driver of respiratory disease, making reverse causality and residual confounding difficult to exclude [132, 137]. Marked interindividual variability in the neonatal microbiota, together with differences in probiotic formulations and dosing across studies, particularly in extremely preterm infants, further constrains the immediate clinical translation of microbiota-targeted therapies [137, 139, 140].

Mechanistic insights are predominantly derived from animal models, which allow causal relationships to be explored under controlled experimental conditions but may not fully recapitulate the complexity of human neonatal physiology, and direct mechanistic evidence linking gut microbiota-derived signals to airway epithelial or immune cell function in human neonates specifically remains scarce [12, 40, 132, 141]. Another important limitation is the predominance of bacterial community profiling using 16S rRNA sequencing, whereas fungal, viral and other nonbacterial components of the microbiome, as well as functional multi-omics approaches integrating metagenomics, metabolomics and host immune profiling, remain comparatively underexplored [16, 17, 44, 45]. Many earlier studies relied on single-time-point or infrequent sampling, which may not capture the dynamic nature of early-life microbiome development, particularly during acute illness; more recent longitudinal designs with dense sampling have begun to address this gap [15, 104, 137, 142].

Future well-designed longitudinal studies and randomised interventional trials incorporating standardised sampling strategies and integrated multi-omics analyses will be essential to distinguish association from causation and to determine whether microbiome-targeted interventions can meaningfully improve respiratory outcomes in early life.

## Clinical implications and translational opportunities

The growing recognition of the gut–lung axis as a candidate contributor to neonatal and later childhood morbidity provides promising opportunities for improving neonatal respiratory care through microbiome-informed approaches. However, dysbiosis at either site is independently associated with neonatal morbidity and this alone does not prove a true interaction between the two compartments. Antibiotic therapy is a key confounder. Broad-spectrum antibiotics in unwell infants are given to control infection at one site but inevitably affect microbial communities elsewhere, which complicates any causal interpretation [143]. From a clinical perspective, the gut–lung axis offers a framework for biomarker discovery, risk stratification and the identification of novel therapeutic targets that extend beyond the lung itself [90, 94, 102, 117–119, 144].

Several longitudinal studies have reported associations between reduced microbial diversity, increased abundance of *Proteobacteria*, altered microbial metabolic pathways and subsequent development of BPD, suggesting that combined microbiome–metabolome profiling may support early risk stratification in vulnerable preterm infants [28, 90]. Together, these findings suggest that combined microbiome–metabolome signatures may enable early identification of infants at high risk for adverse pulmonary outcomes, thereby opening a window for timely preventive interventions during critical periods of immune and lung development.

Among interventional strategies, probiotic and prebiotic supplementation remains the most extensively studied approach to modulate the neonatal gut microbiota [94, 117–119, 144]. Meta-analyses consistently demonstrate that administration of *Bifidobacterium* and *Lactobacillus* species reduces the incidence of NEC and all-cause mortality in preterm infants [145]. Although evidence for a direct protective effect against LONS and BPD is inconsistent, attenuation of systemic inflammation and modulation of immune maturation *via* the gut may indirectly confer pulmonary benefit [24, 25, 29, 94]. These observations support the concept that gut-targeted interventions may influence respiratory outcomes even in the absence of lung-specific treatment effects.

Beyond conventional probiotics, emerging approaches including strain-specific live biotherapeutics, postbiotics and microbiome-derived metabolites are being explored to more precisely modulate host immune responses [146]. Recent preclinical studies also suggest that manipulation of the intestinal mycobiome may influence hyperoxia-induced lung injury, further expanding the spectrum of potential microbiome-targeted interventions [26]. Additionally, bioactive components of human milk, including HMOs and S100A8/A9, promote colonisation by beneficial taxa, enhance epithelial barrier function and support immune tolerance, making them a particularly attractive and translationally feasible intervention in the NICU setting [34, 147, 148].

Such approaches may ultimately enable individualised, microbiota-directed strategies to prevent severe respiratory morbidity during the most vulnerable period of early life [131]. Key microbiota-targeted interventions along the neonatal gut–lung axis, ranging from established probiotic strategies to emerging immune- and metabolite-based approaches, are summarised in table 1.

**TABLE 1:** Microbiota-targeted interventions along the gut–lung axis in neonates: clinical end-points, evidence and key references

Intervention	Target compartment	Proposed mechanisms	Clinical end-points studied	Level of evidence	Translational considerations	Key references
**Conventional probiotics (*Bifidobacterium*, *Lactobacillus*)**	Gut microbiota	Promotion of beneficial taxa, suppression of pathobionts, modulation of systemic inflammation and immune maturation	NEC incidence, all-cause mortality; inconsistent effects on LONS and BPD	High (RCTs, meta-analyses)	Strain- and dose-dependent effects; product heterogeneity; safety considerations in extremely preterm infants	[39, 139, 144, 145]
**Prebiotics/synbiotics**	Gut microbiota	Selective stimulation of commensals; enhanced microbial metabolite production	NEC, sepsis, microbiome composition; limited respiratory end-points	Moderate	Optimal combinations, timing and duration remain unclear	[140, 145, 150]
**Human milk oligosaccharides**	Gut microbiota–immune axis	Enrichment of *Bifidobacterium*, immune tolerance induction, epithelial barrier support	NEC, infection risk; indirect respiratory outcomes	Moderate	High translational feasibility; variability by maternal secretor status and formula supplementation	[151, 152]
**Postbiotics/microbial metabolites (SCFAs, bile acids)**	Immune and epithelial signalling	Regulation of myeloid and T-cell responses; modulation of inflammatory tone	Associations with BPD severity and respiratory morbidity	Low–moderate	Promising for precision approaches; safety and dosing in neonates need definition	[12, 60]
**Strain-specific next-generation probiotics**	Gut microbiota	Targeted metabolic or immunomodulatory functions	Preclinical lung injury models; early human studies	Low	Requires functional validation and regulatory clarity	[153, 154]
**S100A8/A9**	Gut microbiota–lung–immune axis	Modulation of innate immune responses; promotion of beneficial taxa, suppression of pathobionts	Preclinical murine sepsis models; LONS and NEC risk in human neonates	Preclinical	Novel target; human neonatal relevance remains to be established	[34, 148]
**Mycobiome modulation**	Gut–lung axis	Fungal–immune interactions affecting inflammatory signalling	Hyperoxia-induced lung injury (animal models)	Preclinical	Novel target; human neonatal relevance remains to be established	[26]
**Multi-omic biomarker-guided interventions**	Gut–lung–immune axis	Integration of microbiome, metabolome, and immune signatures for risk stratification	Prediction of BPD and severe respiratory morbidity	Emerging	Enables precision neonatology; requires longitudinal validation and clinical integration	[15, 89, 127]

BPD: bronchopulmonary dysplasia; LONS: late-onset neonatal sepsis; NEC: necrotising enterocolitis; RCT: randomised controlled trial; S100A8/A9: S100 calcium-binding proteins A8/A9; SCFA: short-chain fatty acid.

## Conclusion

The gut–lung axis has emerged as a clinically relevant framework linking early-life intestinal dysbiosis to pulmonary morbidity in neonates and infants. Dysbiosis not only contributes to NEC and LONS, which influences the trajectory of BPD and may predispose to long-term respiratory disorders, including increased susceptibility to RTIs and asthma [8, 25–27, 110, 149]. Clinical studies suggest that microbiota profiling and metabolomic signatures could serve as early biomarkers to identify infants at highest risk, while preclinical and early translational work demonstrates that interventions targeting the gut microbiota may mitigate systemic inflammation and protect the developing lung [26, 65].

Although challenges remain, including heterogeneity of the neonatal microbiome, safety concerns with live microbial therapies and incomplete mechanistic understanding, the integration of microbiome-informed strategies into neonatal care represents a promising avenue for precision medicine. Future research combining longitudinal human cohorts, functional microbiome analyses and targeted interventions will be critical to translate the gut–lung axis into improved short- and long-term outcomes for preterm infants. Ultimately, recognition of the gut–lung axis shifts the paradigm from organ-specific management toward a holistic, systems-level approach to neonatal health.

**Points for clinical practice**

- Early-life gut microbiome composition is a key determinant of immune maturation and is associated with respiratory outcomes, particularly in preterm infants.
- Intestinal dysbiosis is associated with increased risk of BPD, NEC, LONS and RTIs, and may serve as an early risk indicator.
- Antibiotic stewardship and promotion of human milk feeding support healthy microbiota development and should be prioritised in clinical practice.
- Microbiota-targeted interventions (*e.g.* probiotics) show clear benefits for NEC, while effects on pulmonary outcomes remain inconsistent.
- The gut–lung axis supports a systemic approach to neonatal care, integrating intestinal and respiratory health in risk stratification and management.
